# Introducing ARTMO’s Machine-Learning Classification Algorithms Toolbox: Application to Plant-Type Detection in a Semi-Steppe Iranian Landscape

**DOI:** 10.3390/rs14184452

**Published:** 2022-09-06

**Authors:** Masoumeh Aghababaei, Ataollah Ebrahimi, Ali Asghar Naghipour, Esmaeil Asadi, Adrián Pérez-Suay, Miguel Morata, Jose Luis Garcia, Juan Pablo Rivera Caicedo, Jochem Verrelst

**Affiliations:** 1 Department of Range and Watershed Management, Faculty of Natural Resources and Earth Sciences, Shahrekord University, Shahrekord 8818634141, Iran; 2 Image Processing Laboratory (IPL), University of Valencia, C/Catedrático José Beltrán 2, Paterna, 46980 Valencia, Spain; 3 Secretary of Research and Graduate Studies, CONACYT-UAN, Tepic 63155, Mexico

**Keywords:** Automated Radiative Transfer Models Operator, machine-learning classification toolbox, Gaussian process classifier, plant types, Sentinel-2

## Abstract

Accurate plant-type (PT) detection forms an important basis for sustainable land management maintaining biodiversity and ecosystem services. In this sense, Sentinel-2 satellite images of the Copernicus program offer spatial, spectral, temporal, and radiometric characteristics with great potential for mapping and monitoring PTs. In addition, the selection of a best-performing algorithm needs to be considered for obtaining PT classification as accurate as possible. To date, no freely downloadable toolbox exists that brings the diversity of the latest supervised machine-learning classification algorithms (MLCAs) together into a single intuitive user-friendly graphical user interface (GUI). To fill this gap and to facilitate and automate the usage of MLCAs, here we present a novel GUI software package that allows systematically training, validating, and applying pixel-based MLCA models to remote sensing imagery. The so-called MLCA toolbox has been integrated within ARTMO’s software framework developed in Matlab which implements most of the state-of-the-art methods in the machine learning community. To demonstrate its utility, we chose a heterogeneous case study scene, a landscape in Southwest Iran to map PTs. In this area, four main PTs were identified, consisting of shrub land, grass land, semi-shrub land, and shrub land–grass land vegetation. Having developed 21 MLCAs using the same training and validation, datasets led to varying accuracy results. Gaussian process classifier (GPC) was validated as the top-performing classifier, with an overall accuracy (OA) of 90%. GPC follows a Laplace approximation to the Gaussian likelihood under the supervised classification framework, emerging as a very competitive alternative to common MLCAs. Random forests resulted in the second-best performance with an OA of 86%. Two other types of ensemble-learning algorithms, i.e., tree-ensemble learning (bagging) and decision tree (with error-correcting output codes), yielded an OA of 83% and 82%, respectively. Following, thirteen classifiers reported OA between 70% and 80%, and the remaining four classifiers reported an OA below 70%. We conclude that GPC substantially outperformed all classifiers, and thus, provides enormous potential for the classification of a diversity of land-cover types. In addition, its probabilistic formulation provides valuable band ranking information, as well as associated predictive variance at a pixel level. Nevertheless, as these are supervised (data-driven) classifiers, performances depend on the entered training data, meaning that an assessment of all MLCAs is crucial for any application. Our analysis demonstrated the efficacy of ARTMO’s MLCA toolbox for an automated evaluation of the classifiers and subsequent thematic mapping.

## 1 Introduction

Satellite images provide valuable geospatial data for monitoring land surface conditions [1] and both characterizing and mapping land use/land cover [2]. The concepts of ‘land cover’ and ‘land use’ are commonly confused in most land surveys including those derived from satellite imagery, although they are fundamentally distinct [3]. Land use is a socio-economic interpretation of the activities that take place on the earth’s surface [4]. Hence, the goal of land-use classification is to assign a land-use label to larger spatial entities, which form a functional unit. Instead, land-cover classification focuses on the assignment of class labels to (frequently small) image sites [5]. Thus, land-cover classification involves the discrimination of land-cover types through different classification methods, which were developed in the field of remote sensing [6]. Land cover mapping plays an important role in studies related to changes in environmental conditions, climate, biodiversity [7], conservation and management of landscapes [8], and ecosystem services [9]. Plant communities are considered the fundamental unit of natural habitats [10], and plant-types (PTs) are the representative plant communities at a site [11]. PTs are distinctive kinds of land cover that differ from other kinds of land in the ability to produce distinctive types and amounts of vegetation and respond alike to management actions and natural disturbances.

Land cover assessment and classification, whereby each image pixel is assigned to a class label indicating the physical material of the surface, are nowadays standard tasks in satellite image processing [12–14]. Although Earth observation is used to land cover assessment, its application for vegetation-type mapping has been mainly limited to structural classification units such as cropland [15], mixed forest [16], or woodland [8]. At the same time, the distinction of PTs remains challenging [17], particularly in a heterogeneous landscape [18]. Sub-classes of a land-cover unit such as PTs have similar spectral behavior (low inter-class separability) and a complex spatial structure on the landscape [19]; therefore, these heterogeneous vegetation communities remain challenging to classify using satellite imagery. Understanding the weaknesses and strengths of various remotely sensed data is the first essential step for the selection of appropriate satellite images and creating successful thematic maps.

Traditionally, the Landsat satellite series have provided a long record and valuable datasets for land cover monitoring and mapping due to their free availability and regular revisit capabilities [20]. However, studies have also noted that Landsat sensors easily misrepresent the spatial variations of vegetation species and are inadequate for highly heterogeneous landscapes, due to a spatial resolution of 30 m [21–24]. Finer spatial resolution multispectral sensors are required for more detailed characterization of vegetation species [25]. In 2015, the Sentinel-2A (S2) satellite was launched for data continuity and enhancement of Landsat performance, followed by the launch of S2B in 2017. S2 is equipped with a multispectral sensor of high and medium spatial resolution (10, 20, and 60 m) and 13 spectral bands [26]. Given its high spatial and temporal resolution and excellent radiometric characteristics, this satellite suits perfectly for vegetation monitoring and mapping.

Because each of the classifiers interprets data in a different way, usually leading to varying mapping results, the selection of a best-performing algorithm needs to be considered for the classification task [27]. With the ongoing gain in computational power, attractive pixel-based supervised methods have been developed for efficient and accurate land-cover mapping [28]. Especially, the family of machine-learning classification algorithms (MLCAs) emerged as a powerful non-parametric approach for classification tasks. MLCAs have the potential to generate adaptive, robust relationships and, once trained, they are fast to apply [29]. Machine learning methods cope typically well with the strong nonlinearity of the functional dependence between the label and the acquired reflectance data. Such models can substantially reduce the time and cost of constructing accurate land-cover maps [19,30,31]. Popular pixel-based supervised MLCAs can be categorized according to families of: (1) neural networks, (2) decision trees, (3) kernel-based algorithms, or (4) ensemble learning. However, these categories are merely semantics, and in reality, all kinds of cross-category classification methods exist, e.g., random forests, which is an ensemble decision tree classifier. For each of these families, algorithms diversified in different directions and variations; each of these methods have strengths and weaknesses in terms of accuracy, processing time, and easiness to use [32,33]. While accuracy is considered the most important criterion, apart from the applied classifier in supervised methods, the quality of the generated map depends on the representativeness of the training data as they were theoretically proven [34,35]. Despite the availability of a diverse array of methods in distinct computing languages, there is a need for an intuitive software toolbox that enables fast and easy testing of advanced methods to obtain a systematic and comprehensive evaluation. After all, one can only be certain of using the best-performing classifier when all of them were tested and compared.

Moreover, for the broader remote sensing community, MLCAs may be perceived as complicated algorithms. Supervised machine learning algorithms possess several algorithm options and parameters to be tuned, so-called hyperparameters, which typically require certain expertise and the know-how of a programming language. When alternatively inspecting available software packages, to the best of our knowledge, no freely downloadable toolbox brings the diversity of pixel-based MLCAs together into a single intuitive user-friendly graphical user interface (GUI). To fill this gap and to facilitate and automate the usage of MLCAs, in this study, we present a novel software package that allows systematically training, analyzing, and applying supervised MLCA models. The so-called MLCA toolbox has been implemented within the in-house developed toolbox called ARTMO (Automated Radiative Transfer Models Operator). ARTMO is a scientific GUI toolbox originally dedicated to the running of radiative transfer models and the retrieval of vegetation properties from optical imagery [36]. Over the years, the software package has been expanded with all kinds of image processing tools and toolboxes (e.g., [37–39]), with now also the MLCA toolbox.

Given the available diversity of advanced classifiers, the impact of distinct pixelbased ML classifiers in interpreting heterogeneous vegetation communities with similar spectral features has not yet been quantitatively assessed, and remains a challenge. In this study, we aim to bridge this gap with the algorithms and versatility offered by the MLCA toolbox. At the same time, we aimed to provide a quantitative assessment of the implemented algorithms for landscape monitoring and assessment. To this end, we chose a heterogeneous rangeland landscape in Southwest Iran as a demonstration case. It is a challenging semi-steppe region for vegetation classification as PTs are overlapping and spectrally alike. Altogether, the overarching objectives of this study are: (1) to present the novel MLCA software toolbox for semi-automatically analyzing MLCAs and classification tasks; (2) to evaluate the implemented MLCAs on their performance and robustness; and (3) to apply the best-performing MLCAs to remote sensing imagery to test the robustness and accuracy in real scenarios.

## 2 Materials and Methods

### 2.1 ARTMO Toolbox

**Figure 1:**
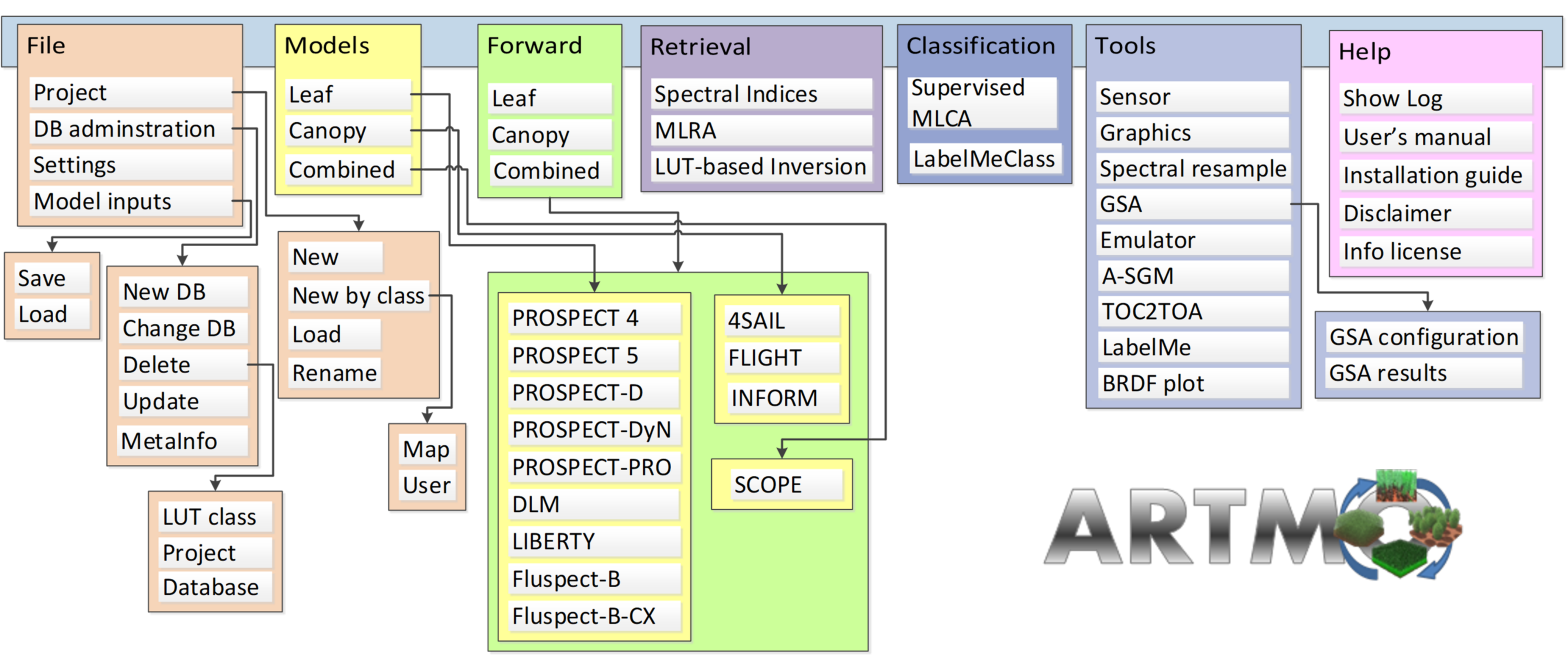
Schematic overview of ARTMO’s v3.29 modules (RTMs, toolboxes, tools).

First, a brief introduction of ARTMO is given, followed by the specifics of the MLCA toolbox. ARTMO is a modular GUI toolbox developed in Matlab, with the original purpose to automate the simulation of radiative transfer models (RTMs) [40]. ARTMO brings multiple leaf and canopy RTMs together with essential tools required for a semi-automatic retrieval of biophysical and biochemical variables in one GUI toolbox. ARTMO has been expanded over the years with all kinds of RTMs and image processing toolboxes, such as mapping, emulation, sensitivity analysis, and scene generation. ARTMO is linked with a relational SQL database management system (MySQL, version 5.5 or 5.6; local installation required), where all generated data (i.e., simulations, statistical results) and trained models are stored along with metadata, thus, allowing the re-running of earlier models or simulations. Figure 1 presents ARTMO v3.29’s main window and a systematic overview of the drop-down menu below. See also http://artmotoolbox.com/ (accessed on 6 September 2022) for details about each of the implemented RTMs, toolboxes, and tools.

This paper introduces ARTMO’s classification “MLCA toolbox” and the accompanying “LabelMeClass” tool, which facilitates the generation of labeled data. The key features of the MLCA toolbox are outlined below, starting with an overview of included classifiers.

### 2.2 ARTMO’s Machine-Learning Classification Algorithms (MLCA) Toolbox: Classifiers

In this first official version (v.1.1) of the MLCA toolbox, 19 supervised MLCAs have been implemented belonging to the key families of supervised classifiers, most of them belonging to families of machine learning methods. It must be remarked that this first version is restricted to pixel-based classifiers, implying that object-based sub-pixel-based or scene-based deep learning classifiers have not been considered yet. However, pixel-based classifiers allow to learn and characterize complex spectra and fit decision functions that are able to predict full scenes acquired by satellites.

Pixel-based classifiers are traditionally categorized into either parametric or non-parametric methods (see book [41] and reviews [32,42]). Parametric methods are based on probabilistic theories, modeling the decision boundaries between classes from a fixed number of parameters, independent of the number of samples, using global criteria for the classification [43]. Conversely, the non-parametric methods guide the grouping of classes based on the digital number (single band/image) or spectral reflectance (multispectral image) and other characteristics, such as shape and textural attributes of the scene. The distribution of the image values is independent and it is focused on the local data structure, requiring a high set of samples for the classification process [44].

The following parametric classifiers are implemented: (1) discriminant analysis, and (2) naive Bayes. See also Table 1 for a brief description and references. Apart from the parametric classifiers, a diversity of non-parametric classifiers have been implemented, most of them due to the available Matlab “Statistics and Machine Learning Toolbox” and “Deep Learning Toolbox”, but also external codes, such as Gaussian process classification coming from the “simpleClass” repository (https://github.com/IPL-UV/simpleClass, accessed on 6 September 2022). According to the logic of loaded libraries, the implemented non-parametric classifiers can be categorized as follows: (1) nearest neighbor, (2) decision trees, (3) error-correcting output codes (ECOC), (4) ensemble learners, (5) neural networks, and (6) Gaussian processes. These categories are briefly described in Table 2. All classifiers were provided in their default settings, unless otherwise specified. Algorithm settings can be changed within Settings -> Advanced settings.

### 2.3 MLCA Toolbox: Workflow

**Figure 2:**
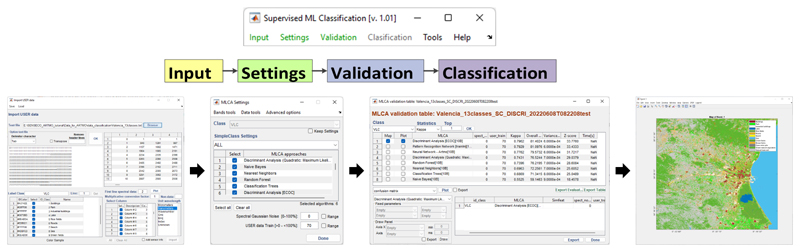
Schematic overview of ARTMO’s MLCA toolbox. The toolbox is on top, and the main GUIs are underneath.

The following step addresses the analysis of multiple MLCA-based classification strategies within the MLCA toolbox. The toolbox and its workflow logic is illustrated in Figure 2. The key modules and their key characteristics are outlined next.

The toolbox operates by starting with inserting input data required for training the MLCA algorithms. The input GUI guides the user to import a plain *.txt* file that should consist of labeled data and associated spectra organized as a data matrix. The GUI will guide the user through the data selection steps and check if the data are properly read (not displayed for brevity). Only once the input data are inserted, the “Settings” module can be configured, and only when Settings is configured, the training/validation step can be initiated by giving a name. All model and validation results are stored within MySQL running underneath. Settings include the following options, which can be combined with each of the selected supervised ML classifiers.

#### 2.3.1 Data Splitting and Cross-Validation Options

The training/validation data partition can be controlled by setting the percentage of how much of the labeled data are assigned to training or to validation (i.e., random split-sample approach). Thereby, the user can evaluate the impact of ranging training/validation partitioning by entering a range of training/validation partitions. The classifiers are then trained based on the training data and evaluated based on the validation data. Further, the following cross-validation options are provided whereby all data are used both for training and testing: (1) k-fold cross-validation, (2) hold-out, and (3) leave-one-out. Cross-validation methods may lead to more robust models, and are recommended when having only a limited number of samples at disposal for training and testing. Additionally, the option is provided to load a new external validation dataset. That would allow us to use all input data for training (i.e., 100%), and validation results are then exclusively based on the newly entered dataset.

#### 2.3.2 Dimensionality Reduction, Noise, and Advanced Options

Spectral dimensionality reduction (DR) methods aim to bypass the so-called “curse of dimensionality” (Hughes phenomenon) [56]) that is commonly observed in hyperspectral data, but also can be applicable to multispectral data. With DR methods, the original spectral data are transformed into a lower-dimensional space that allows the definition of a small set of new features (components), which contain the vast majority of the original dataset’s information [57]. As such, it bypasses the need to search for most relevant spectral bands, and thus, simplifies the retrieval problem. Especially in data classification, a diversity of feature extraction and DR methods are available in the literature [58,59]. The most common DR method is provided by the classical principal component analysis (PCA) [60]. PCA has been proven successful in a broad diversity of applications and continues to be the first choice in vegetation properties mapping based on hyperspectral data (e.g., [61–63]). However, situations may occur where PCA is not the best choice and alternatives have to be sought for. As an extension of PCA, partial least squares (PLS) introduce some refinements by looking for projections that maximize the covariance and correlations between spectral information and input variables [64]. Apart from PCA and PLS, in the MLCA toolbox, we introduced nine alternative DR methods into classification, including canonical correlation analysis (CCA), orthonormalized PLS (OPLS), and minimum noise fraction (MNF), as well as their nonlinear extensions derived by means of the theory of reproducing kernel Hilbert spaces. All these methods have earlier been put together into an in-house developed MATLAB library called SIMFEAT [58], which has been included in a GUI in ARTMO’s machine learning regression algorithm (MLRA) retrieval toolbox, and now also in the MLCA toolbox. See [65] for details on the implemented DR methods. Thus, when selecting a DR method, the spectral data will be converted to a user-defined number of components. The classifiers will subsequently use those components for developing classification models. In this way, not only the spectral redundancy is mitigated, but it also speeds up the processing time.

Further, Gaussian noise can be optionally added to the spectral data. The injection of noise is of importance when synthetic (i.e., noise-free) spectra are used for training to account for environmental and instrumental uncertainties, or can be used to assess the robustness of the classifiers given noisy spectra. A range of noise levels can be configured to evaluate multiple noise scenarios.

It is also possible to change the default settings for each of the selected MLCA. The advanced options are available under the drop-down “Advanced options” and are activated when selecting a MLCA. Typically, these setting options are provided by the Matlab codes. It is beyond the scope of this paper to list all the options; they are made accessible with drop-down menus and a button is provided that links to the corresponding Matlab webpage.

#### 2.3.3 Accuracy Metrics and Mapping

**Figure 3:**
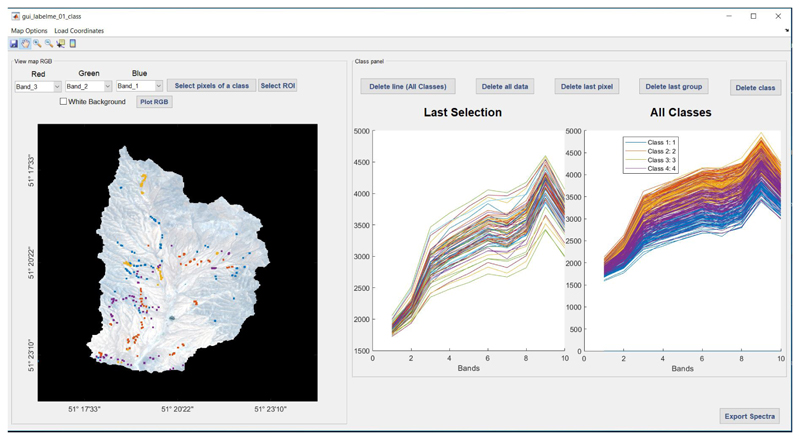
The LabelMeClass tool for extracting labeled data from imagery.

Once the MLCAs are selected and additional options (e.g., dimensionality reduction) are defined in “settings”, the following step is to move towards validation. Hereby, models are trained and then validated against the testing data. The following accuracy metrics are calculated: (1) overall accuracy (OA) and (2) kappa coefficient (k). Additionally, processing time is provided. Results are provided in the validation GUI (see Figure 3). To facilitate the comparison of the implemented classification strategies, the overview table can be sorted based on each of these statistics. Regarding kappa, however, it must be remarked that this metric is not recommended in an accuracy assessment or comparison [66]. We, therefore, decided not to show kappa in the subsequent demonstration study.

For each trained classifier, the following validation statistics can be optionally displayed and exported: accuracy matrix and statistics per class. The statistics per class are the typical accuracy metrics calculated based on the accuracy matrix: (1) producer’s accuracy (also known as recall or true positive or sensitivity), (2) user’s accuracy (also known as precision or positive predictive value), (3) specificity, and (4) F1-Score (%). The F1-Score combines the precision and recall of a classifier into a single metric by taking their harmonic mean, thus, giving a good indicator of the class detection accuracy. See also [67] for details on these metrics. Note also that all models and accuracy metrics are stored within MySQL, and can always be retrieved by selecting “validation -> load”, and then selecting the name of the conducted analysis. Finally, the best-performing model, or any model, can be selected for subsequent mapping purposes. It will then be loaded in the final “Classification” GUI with mapping options.

### 2.4 Extracting Labeled Spectra from Images: LabelMeClass

Along with the release of the MLCA toolbox, we present an associated withol tool called “LabelMeClass”. See also Figure 3. This tool allows to load imagery in ENVI or TIFF format for extracting labeled data, i.e., labels with associated spectra. That file can then serve as training data in the MLCA toolbox. After having loaded an image, the corresponding RGB will appear in the left panel. Subsequently, there are two options to generate labeled training data:

1. Load coordinates based on a *.txt* file consisting of GPS coordinates. The file should consist of class labels and associated coordinates. The tool then checks if the coordinates match within the loaded imagery. It will then extract the associated spectra and visualize the spectra with different colors per class.
2. Manual generation of labeled data. Based on the visualization of the image, pixels can be selected and then assigned to a class. In this way, labeled spectra per class are selected.

For both input data flows, options are added to remove either individual spectra or complete classes. All the labeled spectra will appear in the right panel. Being satisfied with the extracted spectra, finally, the data can be exported to a *.txt* file. Apart from the file with the labeled spectra, an associated *.txt* file is created with metadata, including the data, number of samples, number of bands, name of the classes, information of the geo-data and how the data file is organized. Additionally, as the tool is designed to extract spectral profiles from an image cube, it also provides the option to stack files with individual bands into a single multispectral image.

### 2.5 Demonstration Study: Satellite Data and Feature Selection

A demonstration study is presented next. Concerning the used satellite data, earlier studies already demonstrated that the S2 Multi Spectral Instrument (MSI) imagery is apt for plant community classifications, including heterogeneous landscapes with complex and sparse vegetation patterns [68–70]. S2 offers 13 spectral bands at a spatial resolution of 10, 20 and 60 m. We downloaded a L2A S2 image acquired on 10 June 2020. The image location corresponds to path 164 and row 38 from the USGS (https://earthexplorer.usgs.gov/, accessed on 6 September 2022). This date represents maximum phenological development for the majority of PTs in the study area. The three-coarse resolution bands of 60 m pixel size, i.e., 443 nm (B1), 945 nm (B9), and 1374 nm (B10), were excluded from our analysis, as their purpose is the correction of clouds and atmospheric effects.

First, 20 m spatial resolution bands (bands 5, 6, 7, 8a, 11, and 12) and 10 m spatial resolution bands (bands 2, 3, 4, and 8) were fused through the Gram–Schmidt algorithm. By executing this algorithm, 20 m bands are downscaled to 10 m and a 10 m dataset was created. The pixel size equalizing of all bands when converting the 20 m to 10 m bands was done by resampling other bands with larger pixel sizes using the nearest neighbor algorithm so that the values of adjacent pixels are added for new pixels and avoid the geometric error [71]. Regarding feature selection, selected spectral bands were stacked into a set of datasets and were supplemented with image transformations, namely PCA. From the spectral bands, the first three principal components (PCs) were extracted. These PCs explained > 95% of the data variation and were generated using the spectral information for each image individually [32].

### 2.6 Study Area and Ground Data

**Figure 4:**
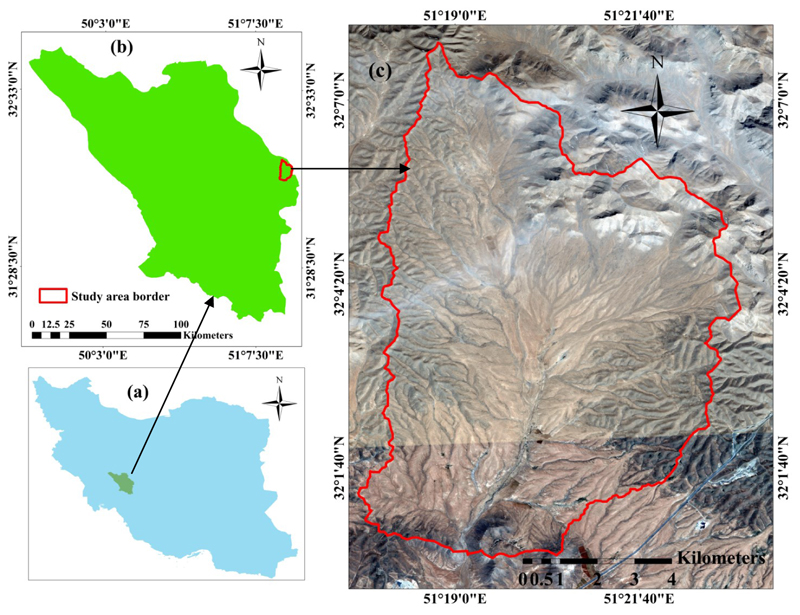
Location of Marjan in the Chaharmahal-Va-Bakhtiari province in southwest Iran: (**a**) Iran border; (**b**) Chaharmahal-Va-Bakhtiari border; (**c**) study area border (Marjan).

The classification study here presented targets the Marjan watershed area located in the Chaharmahal-Va-Bakhtiari province in the southwest of Iran (51°23′12″’E and 32°08′01″N). Before 1990, Marjan was used as an agricultural area with intensive tilling and cultivation. From the year 2000, agricultural activities were reduced due to land abandonment, initiating a nature conservation and rehabilitation program [72]. The vegetation of Marjan went through natural succession, recovering to full canopy cover, with shrubs and perennial grasses becoming dominant. Figure 4 shows the Marjan watershed area with the plant community boundaries, which can be straightforwardly observed due to relatively narrow ecotones and sharp borders. The current vegetation of Marjan consists of shrub land, grass land, semi-shrub land and shrub land–grass land vegetation, while trees are absent. PT data were collected as follows. In spring, which coincides with the beginning growth and maximum plant species growth, we sampled the four identified PTs using three replicates so that the canopy cover was sampled along three transects of 100 m that were evenly distributed throughout the study area with different topography. The sampling was systematic random (the first node was selected systematically but the rest were randomly distributed along the transects). We collected a species-based canopy cover within each quadrat. In each PT, the canopy cover percentage was calculated and the PTs were named according to their dominant floristic composition. For this purpose, first, the dominant plant species of each PT was identified and then its accompanying species was determined by having 50% or more of a canopy cover of a previously dominant species cover. Thus, each PT was named based on a physiognomic–floristic method. Four distinct PTs with the highest coverage in the area were identified, namely: PT1, representing the areas dominated by shrubs, such as *Astragalus verus* Olivier. PT2 represents the areas consisting of grasses, such as *Bromus tomentellus* Boiss. (1846) and *Stipa hohenackeriana* rin. and Rupr. (1842). PT3 involves semi-shrubs vegetation, such as *Scariola orientalis* (Boiss.) Soják (1962) and *Noaea mucronata* (Forsk) Aschers. et Schweinf. (1887). The PT4 class includes shrub-grasses, such as *Astragalus verus* Olivier (1807)—*Bromus tomentellus* Boiss and *Astragalus verus* Olivier (1807)—*Stipa hohenackeriana* Trin and Rupr.

Within each PT, a set of 75 XY random sample points were recorded with a Garmin eTrex 32× Handheld GPS (i.e., 300 points in total). Corresponding spectral data were subsequently added using the LabelMeClass tool. The exported file was then entered into the MLCA toolbox applying a random splitting of 70% for training and 30% for validation.

## 3 Results

**Figure 5:**
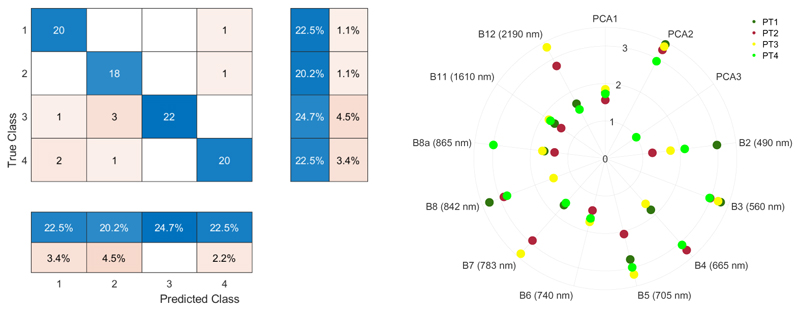
**Left**: confusion matrix of GPC against validation data with correct detection in the blue shade and wrong detection in the red shade. Furthermore, summary percentages per class are provided. **Right**: polar plot of GPC band relevance for the four classes calculated according to the equations described in [74]. The further away from center, the more important.

Building 21 machine-learning classifiers through training and validation with an identical dataset led to varying accuracy results for the classification of the PTs. The MLCA validation module automatically ranked the classifiers based on overall accuracy (OA) results and provided additional accuracy statistics, as shown in Table 3. Results can also be ranked according to the OA. Gaussian process classifier (GPC) was validated as top-performing, with an OA of 90%. The superiority of GPC stands out, as it substantially outperformed all the other classifiers. For this best-performing classifier, the accuracy matrix is exemplarily provided in Figure 5 (left), but note that it can be consulted for any trained model. The second best classifier resulted to be the random forests (RF) algorithm with an OA of 86%. Furthermore, RF delivered top-performing accuracy given that two other types of ensemble-learning algorithms, i.e., tree-ensemble learning (bagging) and decision tree (ECOC), followed at some distance with an OA of 83% and 82%, respectively. The large majority of algorithms closely followed with an OA lowering between 80% and 70%. For instance, the widely used support vector machine classifier produced an OA of 74%. For brevity, the classifiers leading to OA results below 70% were not shown in the table. At the same time, despite the convincing results of the top-performing classifiers, it must be emphasized that the performance and, thus, ranking of these data-driven methods, largely depends on the entered data. Most likely, the validation outcomes of the classifiers are ranked differently in other applications. The message here is that a variability of classifiers are analyzed, so that the best-performing model can be applied to image processing.

Apart from the OA and k accuracy metrics, additional statistical results per class are also provided in Table 3. For each PT, Precision, Sensitivity, Specificity and F1-Score per classifier are given. For instance, for the best-performing GPC, PT3 led to the highest PA with 100%, and PT2 (grass vegetation) led to the lowest PA with 82%. Yet, with only four classes, on the whole, PT class detection accuracies are generally alike, as also expressed by the F1-Score that is around 90%. Furthermore, the second best classifier, RF, leads to a consistent accurate detection of the four PT classes, with an F1 score of around 86%. However, when lowering to the following classifiers, stronger differences between the classes appear, with generally most difficulties in detecting PT class 4.

An interesting property of GPC is the ability to provide band relevance, obtained through automatic relevance determination (ARD) kernels [73]. The relevance of the features can be demonstrated with a polar plot according to Ayala Izurieta et al. [74]. Hereby, the class-specific relevance for each feature is obtained, with values further away from the center expressing increasing importance. The polar plot (Figure 5 (right)) thus provides insights about the key features of the classification process. For instance, it indicates that from the first three PCs, only the second one plays an important role. In fact, the third component had no influence at all, and probably can be discarded. Furthermore, regarding the original S2 bands, B6 (740 nm) and B11 (1610 nm) played only a marginal role. It implies that these two bands expressed too little relevant information to contribute to the identification of the classes. In turn, the most relevant S2 bands appeared to be in the visible green-to-red edge (B3, B4, B5), and then in the near infrared (NIR, B7, B8) and shortwave infrared (SWIR, B12), but only for some classes. In summary, as part of the training process, GPC provides band relevance information to be used to interpret the sensitivity of the spectral information for the performed classification.

**Figure 6:**
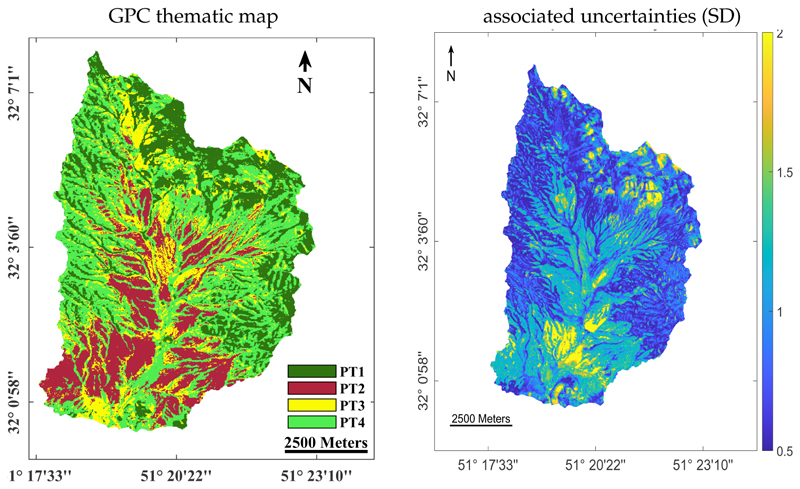
**Left**: thematic map of PTs as obtained from the top-performing Gaussian process classifier (GPC). **Right**: Associated uncertainty map as expressed by standard deviation. The higher the value, the more uncertain.

The seven top-performing models were subsequently applied to the S2 image. Starting with GPC, Figure 6 (left) displays the resulting PT thematic map. Inspecting the GPC-produced PT classes accuracy (Table 3, Figure 5) and obtained map, the following trends are noteworthy. PT1 yielded the highest UA (95.2%), while PT3 led to the lowest UA (84.6%). PT1 is characterized by single-dominated shrubby species with relatively higher canopy cover. Consequently, the higher reflectance of this class, which presents pure pixels of shrubs, resulted in a higher accuracy of classification for this PT. PT3 is characterized by sparse distribution and irregular semi-shrubs species with areas of bare soil frequently visible between plants. Thus, the presence of bare soil and its impact on reflectance received by the satellite sensors likely reduced the classification accuracy in this PT. PT4 consists of two dominant species of shrub-grasses, having dissimilar spectral behavior due to life form differences, therefore, causing more mixed pixels, and consequently, lower UA (86.9%). The GPC-produced map (Figure 6) reveals that PT2 is distributed mainly in the flat areas of the study area, whereas PT1 and PT4 are distributed on more steep slopes. PT3 occurs evenly almost throughout the whole study area. Being a combination of shrub-grass vegetation, PT4 dominates and accounts for 39.8% of the entire study area. PT3 is characterized by sparse distribution and irregular semi-shrubs species with areas of bare soil frequently visible between plants, accounting for only 15.6%. The shrubs vegetation of PT1 and grass vegetation of PT2 accounted for 23.8% and 20.7%, respectively.

Apart from excellent thematic mapping results, another appealing property of the probabilistic GPC is the delivery of associated uncertainty estimates. It is calculated through the predictive variance, which, in the case of a nonlinear GPC with a kernel function *k* over a test pixel x_*_, corresponds to 𝕍[**f**(x_*_)] = **k**(x_*_,x_*_) − **v**⊤ **v**, where **v** is obtained through the Laplace approximation (see Chapter 3, Equation (3.29) in book [55] to obtain the detailed procedure). Although contrarily to regression, in the classification framework, the predictive variance loses its physical meaning and provides a confidence measurement about the inferred discrete label. This predictive variance is interpreted as the higher the more uncertain the classification, and conversely, the lower, the more confident. Consequently, the associated uncertainty map (Figure 6 (right)) reveals the fidelity of the classification result. A systematic spatial pattern appears with higher uncertainties in the valleys and patches on mountain tops. Practically, the uncertainty map illustrates which regions are more or less well represented in the training dataset. Hence, the map informs where additional samples could be collected and added to the training dataset to increase the robustness of the classification model.

**Figure 7:**
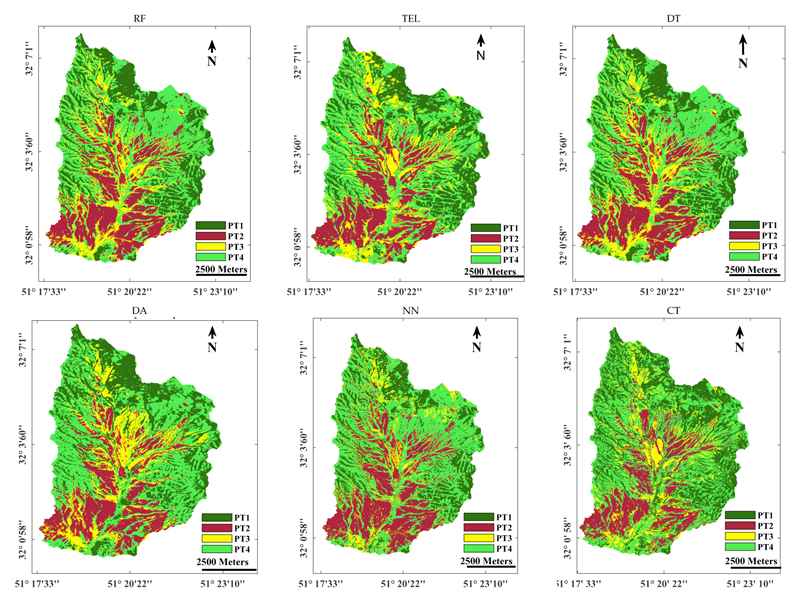
Thematic maps of PTs as obtained from the second- to seventh-best validated classifiers (see Table 3). RF: random forests, TEL: tree-ensemble learning (bag), DT: decision tree (ECOC), DA: discriminant analysis (ECOC), NN: neural network (Adam), CT: classification trees.

In comparison, generated maps, as produced by the subsequent six best-performing classifiers, are shown in Figure 7, being: (1) RF, (2) tree EL (bag), (3) decision tree (ECOC), (4) discriminant analysis (ECOC), (5) neural networks, and (6) classification trees. The spatial differences among the maps are evident, with RF resembling most closely the top-performing GPC map. Consequently, the revealed spatial patterns and differences highlight the relevance of striving for applying the most accurate classifier. Inspecting accuracy results alone is not enough; although overall accuracies of multiple classifiers may be alike given a limited validation dataset, the impact of the classifiers on thematic mapping and resulting spatial patterns over larger areas can be substantial. Hence, apart from the importance of applying the top-validated classifier, a thorough visual inspection remains critical to ensure the produced map is fully accurate.

## 4 Discussion

Accurate thematic maps that depict the patterning of land cover in the landscape are indispensable for land cover conservation and effective managing [75]. This accuracy is not only dependent on the classification scene or the data themselves, but it is also strongly bound to the applied classification method. For that purpose, this study aimed to introduce ARTMO’s new classification “MLCA toolbox” with an application of PTs mapping in a heterogeneous, semi-steppe Iranian landscape. The particular challenge here lies in the spectral similarity of the PTs, being vegetation sub-classes. The classification layout used for separating PTs is based on the premise that each class is the result of a distinct combination of diagnostic species. We selected four dominant PT classes in the study area: PT1 with the shrubs vegetation, PT2 with the grass vegetation, PT3 with the semi-shrubs vegetation, and PT4 with the combination of shrub-grass vegetation. Aggregating precise information of related PTs characterized by rather similar spectral responses at the landscape level is an ambitious classification task. Apart from the applied classifier, the success of land-cover mapping in general and PTs specifically relies on the careful selection of remote sensing data with appropriate spatial and spectral features to improve classification accuracy [76]. The present era of Earth observation with multiple optical satellite sensors orbiting the Earth has provided free imagery with high spectral, spatial, and temporal resolutions considered essential tools for vegetation cover mapping. Here, S2 satellite imagery was selected for the classification task. S2 imagery has demonstrated its utility for vegetation mapping due to its optimal radiometric resolution [77]. Typically, the most sensitive bands in vegetation mapping studies are located in the visible red, red edge, and NIR domains. Since the MSI onboard the S2 satellite is equipped with bands located in the visible red and red edge (B4, B5, B6, and B7), NIR (B8 and B8a) and even SWIR (B11, B12), it allows to retrieve valuable information related to vegetation properties, e.g., to discriminate PTs [78–80]. Regarding the extraction of labeled spectra for training or validating the classifiers, ARTMO’s new LabelMeClass tool facilitates the collection of spectra from a loaded image, either based on GPS coordinates or visually selected. The export option prepares the output *.txt* file ready-to-import into the MLCA toolbox.

### 4.1 Selection of the Best Machine-Learning Classification Algorithm

After the collection of labeled spectra for training and validation, the next essential step was selecting a suitable classification algorithm to obtain an accurate thematic map. This depends on the capacity of the supervised algorithm to classify land covers correctly, its operational capacity, interpretability and transparency, given the entered training data. The current generation of machine-learning classifiers proved to be more accurate and robust than conventional classification techniques in multiple studies, especially when the feature space is complex and data present different statistical distributions [81–83]. Its popularity in mapping applications lies in multiple factors [81,83–86]: (1) the ability of these techniques to learn complex patterns, nonlinear in many cases; (2) the high generalization capacity of these algorithms, which allows to apply them on incomplete or noisy databases; (3) the possibility of incorporating a priori information; and above all, (4) their independence with respect to the data statistical distribution. This latter characteristic makes it possible to incorporate data from different sensors, auxiliary variables—such as those derived from digital terrain models—or even categorical variables [87,88].

Despite the popularity of common supervised machine-learning classifiers in thematic mapping applications, a freely downloadable software package that brings the diversity of the latest MLCAs together in a streamlined toolbox was still missing. To facilitate the use of these advanced algorithms by the broader community, here we introduced a novel GUI toolbox within ARTMO’s software framework that guides the user through evaluating over 20 supervised MLCAs and applying a selected model for mapping applications. The MLCA toolbox uses the same logic as ARTMO’s machine learning regression toolbox [89], i.e., it streamlines the training and validation of the classifiers, and the user can then select a classification model (e.g., the best-performing one) for mapping applications. Furthermore, the toolbox largely uses the same machine learning algorithms available from Matlab, but then the classification version. Finally, all models and validation results are being stored in a MySQL server running underneath, ensuring easy loading and application of an earlier model.

### 4.2 Perspectives of Gaussian Process Classifier (GPC) in Remote Sensing

Based on the evaluation of all included MLCAs using the collected semi-steppe PTs dataset, GPC was identified as a top-performing classifier with an OA of 90%. GPC out-performed widely used MLCAs such as RF, support vector regression, or neural networks. Each of these classifiers were evaluated as top-performing against other common classifiers in earlier studies [30,90,91]. The outstanding accuracy reached by GPC is remarkable; as in this study, we did not aim for detecting the usual thematic land covers with distinct spectral behaviors (e.g., water, land, vegetation). Instead, we targeted the detection of related PTs where the spectral behavior is alike, leading classical classifiers to fail PTs detection with adequate accuracy [92]. In addition, the majority of past studies dedicated to thematic mapping using satellite imagery evaluated only a few classifiers [42,84]. To the best of our knowledge, this is the first time that more than 20 different MLCAs were evaluated.

GPC has not yet received the full attention it deserves [93]. As pointed out in this work, GPC comes from a probabilistic framework which offers a bunch of advances with respect to other techniques inside the statistical learning theory. These include the capability of providing confidence estimation about the inferred class value (i.e., uncertainty map), the optimization of hyperparameters under an optimization framework based on the maximization of the likelihood function, and the capability of optimizing more complex kernel functions. We used the ARD [73] kernel whose complexity increases with the number of input dimensions or, equivalently, with the number of wavelengths. The advantage of the ARD kernel is that it provides band ranking information, and thus, identifies the most sensitive bands in the development of the classification model. The advantage of uncertainty information is that it allows inspecting how the GPC model performs over the complete image. Practically, high uncertainty over an area tells that the model would benefit from additional sampling over that area. At the same time, uncertainty can be used to assess the portability of the model when applied to other images in space and time [94]. On the downside, GPCs are computationally demanding; the optimization process involves many matrix inversions and parameter choices. Despite this, the framework in which GPCs are built is robust, allowing to obtain optimal estimates with a relatively small number of samples. When the training dataset is fairly small, as presented in this study (i.e., 300), then training time is in the order of seconds (12 s), and mapping runtime is quasi-instant (here <1 min). When aiming to use larger training datasets (i.e., in the order of thousands), then alternative solutions may have to be introduced as presented in the literature, such as a sparse-approximation, for example, informative vector machine [93], active learning [95], or Fourier approximations [96]. These studies indicate that variations of GPCs have been developed, e.g., in order to cope with larger training datasets, and are of interest to be implemented into future versions of the MLCA toolbox.

As a closing remark, it must be mentioned that the MLCA toolbox does not have to be restricted to the processing of multi-spectral data. When combining the classifiers with the dimensionality reduction methods (e.g., PCA), it is perfectly prepared to process hyperspectral data. Within the emerging spaceborne imaging spectroscopy era with the recently launched Environmental Mapping and Analysis Program (EnMAP), PRecursore IperSpettrale della Missione Applicativa (PRISMA), and planned operational hyperspectral satellites, it is expected that the toolbox will open opportunities for improved or new thematic mapping applications. At the same time, we envision to expand the MLCA toolbox with the latest machine learning algorithms, including in the field of image-based deep learning, as well as in the field of unsupervised learning. Furthermore, a change detection tool is foreseen. The ARTMO software framework is freely downloadable at: http://artmotoolbox.com/ (accessed on 6 September 2022).

## 5 Conclusions

Machine learning algorithms became standard practice in the field of vegetation classification. However, despite the diversity of distinct classifiers implemented in popular computing languages, an intuitive software GUI toolbox that enables automated evaluation of multiple MLCAs was still lacking. In this study, we introduced the ARTMO’s MLCA toolbox encompassing 21 supervised MLCAs that belong to the key families of supervised per-pixel machine-learning classification algorithms, including decision trees, neural networks, ensemble methods, and kernel-based classifiers.

To demonstrate the utility of the MLCA toolbox, a vegetation thematic mapping study was conducted, focusing on the detection of plant-types. PTs in a semi-steppe Iranian landscape, consisting of a few dominant plant species, are characterized by a complex spatial structure and are often spectrally similar, leading to a low inter-class separability. Therefore, these heterogeneous vegetation communities are challenging to aggregate using satellite imagery and conventional classifiers. Based on a dataset collected from four dominant PTs, a Gaussian process classifier (GPC) excelled with an OA of 90%. Moreover, the GPC not only outperformed established MLCAs, such as random forests and neural networks for this challenging task, but also provided band ranking information and associated uncertainty estimates. Finally, the MLCA toolbox has been made freely available to the community, allowing the evaluation of the MLCAs for any thematic mapping application from optical Earth observation data.

## Figures and Tables

**Table 1:** Implemented parametric classifiers into MLCA toolbox.

Classifier	Description	Ref.
Discriminant Analysis (DA)	DA is a linear model for classification and dimensionality reduction, most commonly used for feature extraction in pattern classification problems. First, in 1936, Fisher formulated linear discriminant for two classes, and in 1948, C.R Rao generalized it for multiple classes. LDA projects data from a D dimensional feature space down to a D′ (D > D′) dimensional space in a way to maximize the variability between the classes and reduce the variability within the classes. The quadratic DA is also known as maximum likelihood classification within popular remote sensing software packages.	[45]
Naive Bayes (NB)	The NB is a classification algorithm based on the concept of the Bayes theorem with the “naive” assumption of conditional independence between every pair of features given the value of the class variable.	[46]

**Table 2:** Implemented non-parametric (machine learning) classifiers into MLCA toolbox.

Classifier	Description	Ref.
Nearest neighbor (NN)	The principle behind NN methods is to find a predefined number of training samples closest in distance to the new point, and predict the label from these. The basic NN classification uses uniform weights; that is, the value assigned to a query point is computed from a simple majority vote of the nearest neighbors.	[47]
Decision trees (DT)	Classification trees (CF) fit binary decision tree for multiclass classification. See also: https://es.mathworks.com/help/stats/fitctree.html (accessed on 6 September 2022). Random forests (RF) bags an ensemble of decision trees. Bagging stands for bootstrap aggregation. Every tree in the ensemble is grown on an independently drawn bootstrap replica of input data. See also: https://es.mathworks.com/help/stats/treebagger-class.html (accessed on 6 September 2022). For RF, by default, 100 trees are set as recommended according to [48].	[49,50]
Neural networks (NN)	ANNs in their basic form are essentially fully connected layered structures of artificial neurons (AN). An AN is basically a pointwise nonlinear function (e.g., a sigmoid or Gaussian function) applied to the output of a linear regression. ANs with different neural layers are interconnected with weighted links. The most common ANN structure is a feed-forward ANN, where information flows in a unidirectional forward mode. From the input nodes, data pass hidden nodes (if any) toward the output nodes. The following algorithms have been implemented: *Trainlm* is a network training function that updates weight and bias values according to Levenberg–Marquardt optimization; *Trainscg* is a network training function that updates weight and bias values according to the scaled conjugate gradient method; *Trainbr* is a network training function that updates the weight and bias values according to Levenberg–Marquardt optimization. It minimizes a combination of squared errors and weights, and then determines the correct combination so as to produce a network that generalizes well. The process is called Bayesian regularization. Additionally, the option of developing (per-pixel) deep learning network is added according to Matlab’s deep learning toolbox (https://es.mathworks.com/help/deeplearning/ref/trainnetwork.html (accessed on 6 September 2022)). The NN consists of the following default settings: one hidden layer with 10 neurons, ReLU activation and 10% of dropout. The NN is trained with 1000 epochs using Adam optimization algorithm.	[51,52]
Ensemble learners (EL)	EL combines a set of trained weak learner models and data on which these learners were trained. EL can predict ensemble response for new data by aggregating predictions from its weak learners. The following EL are provided: (1) discriminant EL, (2) k-nearest neighbor (KNN) EL, (3) tree EL (bagging), (4) tree EL (AdaBoost), (5) tree EL (RUSBoost). Bagging and boosting techniques are typically applied to decision trees. Bag generally constructs deep trees. This construction is both time-consuming and memory-intensive. This also leads to relatively slow predictions. Boost algorithms generally use very shallow trees. This construction uses relatively little time or memory. However, for effective predictions, boosted trees might need more ensemble members than bagged trees. See also: https://es.mathworks.com/help/stats/framework-for-ensemble-learning.html (accessed on 6 September 2022)	[50,53]
error-correcting output codes (ECOC)	The ECOC method is a technique that allows a multi-class classification problem to be reframed as multiple binary classification problems, allowing the use of native binary classification models to be used directly. Unlike one-vs-rest and one-vs-one methods that offer a similar solution by dividing a multi-class classification problem into a fixed number of binary classification problems, the error-correcting output codes technique allows each class to be encoded as an arbitrary number of binary classification problems. When an overdetermined representation is used, it allows the extra models to act as “error-correction” predictions that can result in better predictive performance. The following ECOC are provided: (1) discriminant analysis, (2) kernel classification, (3) KNN, (4) linear classification, (5) naive Bayes classification, (6) decision tree, (7) support vector machine. See also https://machinelearningmastery.com/error-correcting-output-codes-ecoc-for-machine-learning/ (accessed on 6 September 2022).	[54]
Gaussian process (GP)	The GP is a stochastic process where each random variable follows a multivariate normal distribution. The goal is to learn mapping from the input data to their corresponding classification label, which can then be used on new, unseen data pixels. When the GP is developed with kernel methods, it allows mapping the original data into a possibly infinite dimensional space in which the input–output relation can be better estimated as it considers more complex and flexible functions than the linear models. As the GP is based on a probabilistic framework, it allows to provide uncertainty estimation per sample. This measurement becomes useful for taking decisions and allows to be more or less confident with the inferred classification label. Moreover, the GP can use more sophisticated kernel functions than the standard linear kernel or the radial basis function (RBF) kernel $k_{RBF}\left(x_{i},x_{j}\right)=\exp\left(-\frac{{\left\|x_{i}-x_{j}\right\|}^{2}}{2\sigma^{2}}\right)$, which can be optimally tuned through the likelihood maximization. In the classification case, the output values are discrete (±1); this causes the likelihood function to be non-Gaussian, and then, some approximations should be performed [55]. We choose the Laplace approximation which performs well and is robust. One notable kernel function is the automatic relevance determination (ARD) kernel $k_{ARD}\left(x_{i},x_{j}\right)=\exp\left(-\frac{1}{2}{\left(x_{i}-x_{j}\right)}^{⊤}{\text{Σ}}^{-1}\left(x_{i}-x_{j}\right)\right)$, where Σ is a diagonal matrix whose diagonal tries are constituted by $\left\{\sigma_{1}^{2},\ldots ,\sigma_{d}^{2}\right\}$ parameters to weight each input dimension. This kernel covariance function requires one parameter per input feature; it can be optimized under that framework and it allows to provide a band ranking based on their optimal values. Source code is in: https://github.com/IPL-UV/simpleClass (accessed on 6 September 2022).	[55]

**Table 3:** Accuracy results against validation data for all MLCAs. Results are ordered from best overall accuracy (OA) to worst.

MLCA		PT1	PT2	PT3	PT4
Gaussian processes classifier	Precision (PA %)	86.9	81.8	100	90.9
	Sensitivity (UA %)	95.2	94.7	84.6	86.9
	Specificity (%)	95.5	94.2	100	96.9
	F1-Score (%)	90.9	87.8	91.6	88.8
	OA = 90.0%					
Random forest	Precision (PA %)	86.9	86.3	86.3	86.3
	Sensitivity (UA %)	90.9	86.3	86.3	82.6
	Specificity (%)	95.5	95.5	95.5	95.4
	F1-Score (%)	86.3	86.3	86.3	84.4
	OA = 86.5%					
Tree EL (bag)	Precision (PA %)	91.3	86.3	86.3	68.0
	Sensitivity (UA %)	80.7	82.6	382.6	88.2
	Specificity (%)	96.8	95.4	95.4	90.2
	F1-Score (%)	85.7	84.4	84.4	76.9
	OA = 83.1%					
Decision tree (ECOC)	Precision (PA %)	91.3	75.7	81.8	81.8
	Sensitivity (UA %)	87.5	84.2	72.0	85.7
	Specificity (%)	96.9	91.4	93.7	94.0
	F1-Score (%)	89.3	78.0	76.6	83.7
	OA = 82.0%					
Discriminant analysis (ECOC)	Precision (PA %)	86.9	86.3	81.8	63.6
	Sensitivity (UA %)	83.3	79.1	85.7	70.0
	Specificity (%)	95.3	95.3	94.0	88.4
	F1-Score (%)	85.1	82.6	83.7	66.6
	OA =79.7%					
Neural network (Adam)	Precision (PA %)	95.6	81.8	72.7	68.1
	Sensitivity (UA %)	81.4	81.0	84.2	71.4
	Specificity (%)	98.3	94.0	91.4	89.7
	F1-Score (%)	88.0	81.0	78.0	69.7
	OA = 79.0%					
Classification trees	Precision (PA %)	91.3	72.7	81.8	68.1
	Sensitivity (UA %)	87.5	80.0	72.0	75.0
	Specificity (%)	96.9	91.3	93.7	89.8
	F1-Score (%)	89.3	76.1	76.6	71.4
	OA = 78.6%					
Discriminant analysis (quadratic)	Precision (PA %)	86.9	72.7	81.8	72.2
	Sensitivity (UA %)	86.9	80.0	69.2	80.0
	Specificity (%)	95.4	91.3	93.6	91.3
	F1-Score (%)	86.9	76.2	75.0	76.1
	OA = 78.6%					
k-nearest neighbors (ECOC)	Precision (PA %)	82.6	63.6	81.8	77.2
	Sensitivity (UA %)	90.4	73.6	69.2	73.9
	Specificity (%)	94.1	88.5	93.6	92.4
	F1-Score (%)	86.3	68.3	75.0	75.5
	OA =76.4%					
Neural network (trainbr)	Precision (PA %)	82.6	68.1	89.3	59.0
	Sensitivity (UA %)	76.0	78.9	79.1	61.9
	Specificity (%)	93.7	90.0	95.3	86.7
	F1-Score (%)	79.1	73.1	82.6	60.4
	OA = 74.1%					
Support vector machines (ECOC)	Precision (PA %)	86.9	68.1	77.2	63.6
	Sensitivity (UA %)	80.0	71.4	68.0	77.7
	Specificity (%)	95.3	89.7	92.1	88.7
	F1-Score (%)	83.3	69.7	72.3	70.0
	OA = 74.1%					
Linear classification (ECOC)	Precision (PA %)	86.9	68.1	77.2	36.3
	Sensitivity (UA %)	80.0	71.4	68.0	77.7
	Specificity (%)	95.3	89.7	92.1	88.7
	F1-Score (%)	83.3	69.7	72.3	70.0
	OA = 74.0%					
Neural network (trainscg)	Precision (PA %)	82.6	72.7	72.7	63.6
	Sensitivity (UA %)	86.3	69.5	66.6	70.0
	Specificity (%)	94.0	90.9	90.7	88.4
	F1-Score (%)	84.4	71.1	69.5	66.6
	OA = 73.0%					
Naive Bayes	Precision (PA %)	78.2	90.9	45.4	72.7
	Sensitivity (UA %)	81.8	76.9	90.9	53.3
	Specificity (%)	92.5	96.8	84.6	89.8
	F1-Score (%)	80.0	83.3	60.6	61.5
	OA = 72.0%					
Neural network (trainlm)	Precision (PA %)	82.6	63.6	77.2	63.6
	Sensitivity (UA %)	82.6	77.7	60.7	70.0
	Specificity (%)	93.9	88.8	91.8	88.4
	F1-Score (%)	82.6	70.0	68.0	66.6
	OA = 72.0%					
Tree EL (AdaBoost)	Precision (PA %)	86.9	95.4	45.4	54.5
	Sensitivity (UA %)	83.3	61.7	62.5	80.0
	Specificity (%)	95.3	98.1	83.5	86.4
	F1-Score (%)	85.0	75.0	52.6	64.8
	OA = 70.7%					
Discriminant EL	Precision (PA %)	86.9	90.9	36.3	63.6
	Sensitivity (UA %)	76.9	71.4	88.8	53.8
	Specificity (%)	95.2	96.7	82.5	87.3
	F1-Score (%)	81.6	80.0	51.6	58.3
	OA = 69.6%					

## Data Availability

Not applicable.
